# Aromatase in the Human Brain

**DOI:** 10.1089/andro.2021.0007

**Published:** 2021-12-23

**Authors:** Iñigo Azcoitia, Pablo Mendez, Luis M. Garcia-Segura

**Affiliations:** ^1^ Department of Cell Biology, Faculty of Biology, Universidad Complutense de Madrid and Centro de Investigación Biomédica en Red de Fragilidad y Envejecimiento Saludable (CIBERFES), Instituto de Salud Carlos III, Madrid, Spain.; ^2^ Instituto Cajal, Consejo Superior de Investigaciones Científicas (CSIC), Madrid, Spain.

**Keywords:** aging, amygdala, cerebral cortex, hippocampus, hypothalamus, thalamus

## Abstract

The aromatase cytochrome P450 (P450arom) enzyme, or estrogen synthase, which is coded by the *CYP19A1* gene, is widely expressed in a subpopulation of excitatory and inhibitory neurons, astrocytes, and other cell types in the human brain. Experimental studies in laboratory animals indicate a prominent role of brain aromatization of androgens to estrogens in regulating different brain functions. However, the consequences of aromatase expression in the human brain remain poorly understood. Here, we summarize the current knowledge about aromatase expression in the human brain, abundant in the thalamus, amygdala, hypothalamus, cortex, and hippocampus and discuss its role in the regulation of sensory integration, body homeostasis, social behavior, cognition, language, and integrative functions. Since brain aromatase is affected by neurodegenerative conditions and may participate in sex-specific manifestations of autism spectrum disorders, major depressive disorder, multiple sclerosis, stroke, and Alzheimer's disease, we discuss future avenues for research and potential clinical and therapeutic implications of the expression of aromatase in the human brain.

## Introduction

**FIG. 1.**
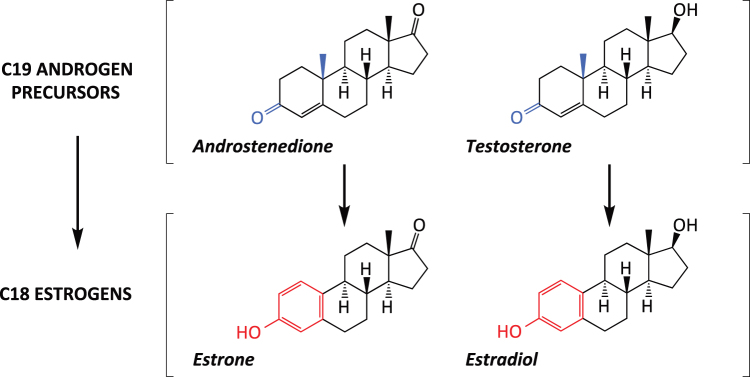
Aromatase cytochrome P450 (P450arom) or estrogen synthase converts androgen c19 precursors in C18 estrogens. For instance, the enzyme converts androst-4-ene-3,17-dione (androstenedione) in estrone and testosterone into estradiol.

Part of the actions of androgens in the body are exerted after their conversion to estrogens by the enzyme aromatase cytochrome P450 (P450arom) or estrogen synthase. The enzyme converts androst-4-ene-3,17-dione (androstenedione) and testosterone into estrone and estradiol, respectively (Fig. 1). Half a century ago, Naftolin and collaborators reported the existence of aromatase activity in samples of human brain tissue isolated from male fetuses.^1,2^ The implication of this finding was that aromatizable androgens can regulate human brain function not only through androgen receptor (AR) but also by the activation of estrogen receptors (ERs) after their conversion to estrogenic metabolites. In addition, although aromatase activity in the brain probably does not affect the overall concentration of androgens, it is plausible that by decreasing the levels of androgens at very specific intracellular domains, aromatase activity may also contribute toward modulate AR signaling in the human brain.

Studies in different vertebrate species, from fish to mammals, have shown that aromatase is expressed in the developing and adult brain and the spinal cord of both males and females. Central aromatase activity participates in a variety of functions that are not restricted to the control of the neuroendocrine axis and the regulation of reproduction or sex differences, but this includes the processing of sensory information, the coordination of sensory inputs with motor outputs, the expression of affective behavior, and the modulation of learning and memory.^3–10^ To exert these actions, estradiol generated by brain aromatase regulates cellular signaling, gene expression, synaptic transmission, and synaptic plasticity,^11–14^ and it is part of the endogenous neuroprotective and anti-inflammatory response activated in neural tissue after injury.^15,16^

Not all of these roles of the enzyme described in animal studies have been ascertained in humans. However, human studies have significantly advanced in recent years and we have considerable new information on the anatomical and cellular distribution of the enzyme in the human central nervous system (CNS), together with new hints on its physiological implication in the neural processing of sensory integration, the modulation of social behavior and cognition, and the central control of body homeostasis. Available data also indicate that brain aromatase expression is altered with aging and under neurodegenerative conditions. Further, genetic and neuropathological findings suggest that the enzyme may participate in the manifestation of brain diseases, including major depressive disorder, autism spectrum disorders (ASDs), and neurodegenerative diseases. Here, we review the available information on the distribution and function of aromatase in the human brain, discussing future avenues for research and potential clinical and therapeutic implications.

## Aromatase Expression and Distribution in the Human Brain

The expression of *CYP19A1*, the aromatase gene, has been detected in the brain of men and women, in structures associated with cognition and memory (frontal cortex, temporal cortex, hippocampus), endocrine and autonomic regulation (hypothalamus), sensorimotor information processing (thalamus, cerebellum), or affective behavior (amygdala).^17–21^ Aromatase protein distribution in these brain regions has been ascertained by immunohistochemical analysis^22^ and positron emission tomography (PET) imaging.^23^ Aromatase messenger RNA (mRNA) and protein expression has been also detected in the human fetal brain, with relative higher levels at the end of the gestational period.^24,25^ In addition, studies performed in the human temporal lobe with different techniques have allowed to demonstrate the coincidence of *CYP19A1* mRNA,^19,26^ aromatase protein,^19^ and aromatase enzymatic activity^27,28^ in the same brain structure. These studies have also shown that men and women have a similar expression, distribution, and activity of aromatase in the brain. Only a small global sex difference, with higher levels in men, has been reported.^29^

The human *CYP19A1* gene contains 9 coding exons that are regulated by at least 10 tissue-specific alternate splicing variants of exon I. Several exon I splicing variants have been identified in the brain of men and women and in human brain cells.^17,19,21,30,31^ These different promoters could be potentially targeted by a large variety of molecules,^17,19,21,32^ allowing a complex regulation of aromatase expression in the human brain. In addition, some of these *CYP19A1*exon I variants have a broad anatomical distribution in the brain, whereas others seem to be restricted to specific brain regions. In agreement with this, aromatase mRNA levels show regional differences, with higher levels in some specific brain structures (thalamus, hypothalamus) compared with others.^17^

Regional differences in brain aromatase levels have been confirmed and expanded by PET studies, which analyze the distribution of binding sites of a labeled aromatase inhibitor and provide a useful comparative estimation of the expression levels of the enzyme in different brain structures. These studies revealed higher expression in the thalamus followed by the amygdala and the hypothalamus/preoptic area and then by the inferior olivary nucleus, accumbens, pons, occipital and temporal cortex, putamen, cerebellum, and the cerebral white matter.^29,33^ The possible function of aromatase in these different regions of the human brain will be discussed later in more detail.

## Cellular Localization

**FIG. 2.**
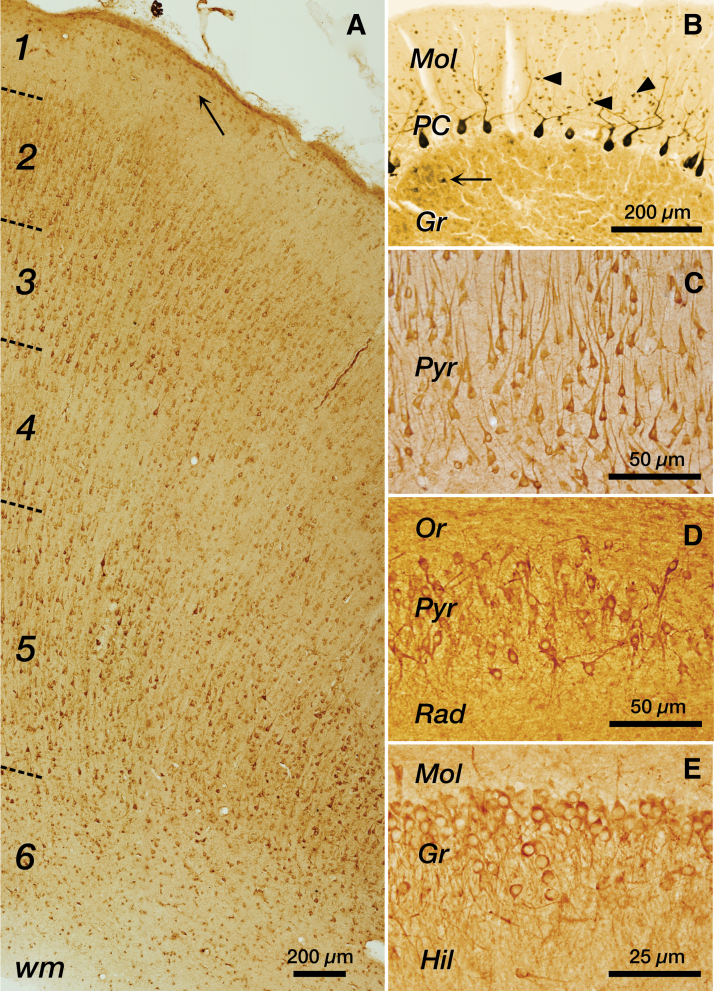
Representative examples of aromatase immunoreactive cells in the human brain. **(A)** Panoramic view of a section of the temporal cortex. Anti-aromatase immunoreactivity is observed in all cortical layers (1–6) and in the wm, but it is particularly intense in pyramidal cells of neocortical layers 2/3 and 5. In layer 1, aromatase immunoreactive cells, most of them with astrocyte morphology, are abundant in proximity of the pial surface (arrow). Female, 63 years old. **(B)** Cerebellar cortex. Intense immunoreactivity is observed in Purkinje neuronal perikarya in the PC and in their dendrites in the Mol, whereas granule cells are not immunoreactive. Immunoreactivity is also observed in interneurons in the molecular layer (arrowheads) and in a few neurons in the Gr (arrow), which may correspond to Golgi neurons. Male, 49 years old. **(C)** Pyramidal neurons in the Pyr of the hippocampal Ammon's horn CA1 region showing aromatase immunoreactivity in the perikaryon and in the basal and apical dendrites. Male, 65 years old. **(D)** Pyramidal neurons in the Pyr of the hippocampal Ammon's horn CA3 region showing aromatase immunoreactivity in the perikaryon and dendrites. Female, 43 years old. **(E)** Hippocampal dentate gyrus showing aromatase immunoreactivity in granule cell neurons in the Gr and a few interneurons in the Hil. Mol, molecular layer of the dentate gyrus. Female, 35 years old. All panels are from immunoperoxidase-stained sections using hydrogen peroxide as substrate and 3,3′-diaminobenzidine tetrahydrochloride as chromogen. In **(B)**, the reaction was intensified by adding a small amount of nickel chloride. Gr, granular layer; Hil, hilus; Mol, molecular layer; Or, Stratum oriens; PC, Purkinje cell layer; Pyr, pyramidal layer; Rad, Stratum radiatum; Wm, white matter.

Neurons are the most abundant cell type expressing aromatase in the human brain (Fig. 2). In human neurons, the enzyme is localized in the perikaryon and in dendrites and axons.^19,22^ The localization of aromatase in these different neuronal compartments suggests that the local production of estradiol by the enzyme may influence a variety of cellular processes, such as signal transduction, gene expression, or synaptic physiology. Aromatase immunoreactivity has been also localized in synaptic terminals of human neurons, including the synaptic vesicles,^34^ in agreement with the proposal that aromatase may generate estradiol in presynaptic terminals that may target ERs in postsynaptic structures.^35^

Although aromatase immunoreactive neurons have been observed in all the regions of the adult human brain examined so far, not all neurons in a given region are immunoreactive for the enzyme (Fig. 2). For instance, in the temporal cortex a high proportion of pyramidal neurons and a small proportion of calbindin and parvalbumin interneurons are immunoreactive for aromatase.^19^ In the hippocampus, pyramidal neurons from CA1 to CA3 are the main neuronal population expressing aromatase, together with some granule cells of the dentate gyrus and specific hilar interneurons that co-express the calcium-binding proteins calbindin, calretinin, or parvalbumin.^20^ In the cerebellum, aromatase immunoreactivity is detected in Purkinje cells, molecular layer interneurons, and a few neurons in the granular layer (Fig. 2).^22^ Aromatase immunoreactive neurons have been also observed in other regions of the human brain, including magnocellular neurons of the supraoptic nucleus of the hypothalamus, the medial mamillary nucleus, the infundibular nucleus, or the basal nucleus of Meynert.^36^ The presence of aromatase in excitatory and inhibitory neurons suggests that estrogen synthesis may play a role in maintaining a balanced interplay between excitatory and inhibitory neuronal activity required for correct brain function.

**FIG. 3.**
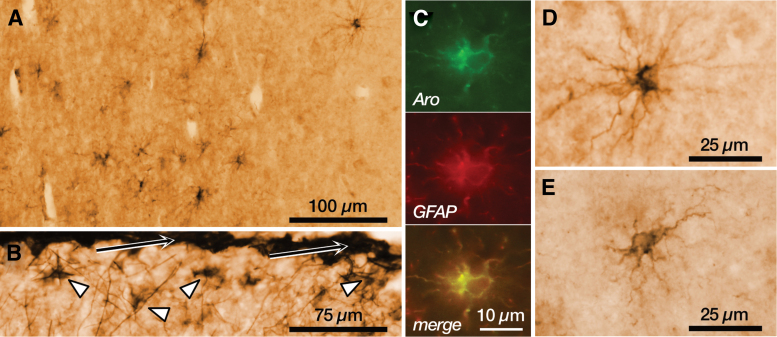
Aromatase immunoreactivity in astrocytes. **(A)** Panoramic view of the cortical white matter in the temporal lobe, showing immunoreactivity in cells with the morphology of fibrous astrocytes. **(B)** Detail of the layer 1 of the temporal cortex showing numerous immunoreactive astrocytes (arrowheads) in the proximity of the pial surface (arrows). **(C)** Immunofluorescence labeling of aromatase (green), the astrocyte cell marker GFAP (red), and the colocalization signal (yellow) in a cortical astrocyte. **(D)** Representative example of a fibrous astrocyte immunoreactive for aromatase in the cortical white matter. **(E)** Representative example of a protoplasmic astrocyte immunoreactive for aromatase in the cortical gray matter. All panels are from a 23-year-old male. GFAP, glial fibrillary acidic protein.

In addition to neurons, a subpopulation of other cells of different types, including fibrous and protoplasmic astrocytes (Fig. 3), oligodendrocytes, some ependymal cells, and choroid plexus cells, show immunoreactivity for aromatase in different human brain regions.^19,20,36–38^

## Physiological and Pathophysiological Function of Brain Aromatase

### Thalamus: sensory integration

Animal research has shown that aromatase is expressed in sensory systems and that local estradiol production by the enzyme modulates the transmission of sensory information,^39–41^ including pain.^42–45^ Aromatase activity is involved in the modulation of sensory function also in humans, because cancer patients treated with aromatase inhibitors often suffer from arthralgia and myalgia^46–48^ and these molecules have been reported to affect the function of sensory systems, such as the retina.^49^ This role of aromatase activity is further supported by the localization of the enzyme by PET^29^ and immunohistochemistry^22^ in the human thalamus, because this highly complex structure is the main sensory information computing brain center. In addition, the thalamus integrates sensorimotor function with high-level cognitive processes. Thus, the thalamus is involved in perception, attention, emotions, memory, language, behavioral flexibility, and the generation of mental representations.^50–53^ Interestingly, aromatase PET signal in the thalamus shows a significant negative correlation with cooperativeness scores in both men and women,^33^ suggesting that aromatase expression in this brain region may be involved in the regulation of estrogen-sensitive thalamo-cortical circuits interpreting sensory information to control social abilities.

### Amygdala and hypothalamus: body homeostasis, social behavior, and major depressive disorder

The amygdala is involved in emotional learning, social behavior, motivation, aggression, and fear and in the processing and representation of emotions. Aromatase levels in the amygdala have been found to be associated with different personality traits. In women, higher aromatase levels in the left amygdala are associated with higher aggression, whereas higher aromatase levels in the right amygdala are negatively associated with novelty seeking and persistence and positively correlated with cooperativeness and self-transcendence.^33^ In contrast, higher aromatase levels in the amygdala are associated with lower verbal and nonverbal cognitive performance in men, but not in women.^54^ Aromatase regulation of androgen/estrogen balance together with sex-specific actions of gonadal hormones in the amygdala may be involved in these functional differences.

In addition, aromatase availability in the amygdala has other functional implications that are similar in both sexes. For instance, aromatase in the amygdala is negatively associated with body mass index in men and women,^55^ in agreement with the association of obesity with negative emotional states and the association of amygdala activity with body mass index.^56^ This function of the amygdala is mediated by its reciprocal connections with the hypothalamus, the main control center of body homeostasis.

The hypothalamus integrates body signals and controls body organs through the autonomic nervous system and by the release of hormones in the neurohypophysis and hormonal-releasing factors in the adenohypophysis. One of the well-characterized roles of the hypothalamus is the regulation of the secretion of gonadotrophins. Studies in female monkeys have shown that aromatase-mediated local estradiol synthesis and action in the hypothalamus is involved in the ovarian positive feedback for the induction of luteinizing hormone surges.^57^ Probably, a similar function of hypothalamic aromatase is operating in women, but this has not yet been studied.

Magnocellular neurons in the human supraoptic and paraventricular hypothalamic nuclei, which produce oxytocin and vasopressin, are immunoreactive for aromatase^36,58^ and this enzyme mediates the effect of testosterone on vasopressin expression by human neuroblastoma cells,^59^ suggesting that locally synthesized aromatized androgen metabolites may be involved in the regulation of magnocellular neurons in the human hypothalamus. The best studied function of vasopressin is blood pressure regulation. However, there is not a clear effect of aromatase inhibitors on blood pressure levels in postmenopausal breast cancer patients.^48^ Still, estradiol generated by aromatase activity in magnocellular neurons may impact other physiological processes regulated by oxytocin and vasopressin, such as reproductive physiology, social bonding, social behavior, anxiety, fear conditioning, and fear extinction.^60^ In this regard, it should be mentioned that a study has found that aromatase levels in the supraoptic nucleus are negatively associated with novelty seeking, reward dependence, and persistence and positively associated with harm avoidance, cooperativeness, and self-transcendence in women.^33^

Aromatase is highly expressed in the hypothalamic paraventricular nucleus of healthy men and women. However, aromatase immunoreactivity is significantly decreased in this hypothalamic region in major depressive disorder patients,^58^ suggesting a possible link of aromatase in the hypothalamus and depression. In this regard, it is relevant to mention that some single-nucleotide *CYP19A1* polymorphisms increase the risk of late-life depression in women without a previous history of major depression, whereas other variants of the gene, associated with higher estradiol levels, decrease the risk of late-life depression in women with a history of major depression.^61^

### Cerebral neocortex and hippocampus: cognition, language, and integrative functions

Aromatase expression in regions of the human brain associated with cognitive function, such as the hippocampus, the prefrontal cortex, and the temporal cortex (Fig. 2),^19–21,27,37,38^ suggest that local conversion of androgens to estrogens participates in the regulation of learning and memory in humans, as it has been demonstrated in animal experimentation studies.^6^ Learning and memory rely on specific patterns of neuronal activity that induce synaptic plasticity. Interestingly, neuronal activity also regulates aromatase activity and estrogen synthesis in the CNS of birds and rodents.^62,63^ However, the role of androgens^64,65^ and estrogens^66–68^ in human cognition is still controversial. Nevertheless, some single-nucleotide *CYP19A1* polymorphisms are associated with decreased performance in memory tests in women^69^ and aromatase inhibitors used for cancer treatment have a negative impact on cognition in some patients,^70^ with subtle effects on hippocampal-dependent verbal and visual memory.^71^ In contrast, treatment with the aromatase inhibitor letrozole does not impair the development of cognitive performance in boys.^72^

Aromatase activity may influence many other cortical functions, in addition to memory and learning. For instance, local production of estradiol by aromatase in the temporal cortex^19,27^ may be involved in visual and auditory integration and language. In this regard, the discovery of single-nucleotide polymorphisms within *CYP19A1* associated with dyslexia categorical traits and with phonological phenotypes is of high relevance.^73^ Interestingly, *cyp19A1* is associated with the control of vocalization in songbirds and teleost fish^74,75^ and *CYP19A1* expression in the human brain correlates with the expression of other dyslexia-associated genes, such as *ROBO1* and *DYX1C1*.^73^

Aromatase activity in associative regions of the occipital, parietal, and frontal lobes may also participate in complex integrative functions, as suggested by the finding that aromatase availability in these cerebral cortex regions is associated with different personality traits in men and women.^33^ These associations have been identified in specific regions of the occipital lobe (lingual gyrus), the parietal lobe (inferior parietal gyrus), and the frontal lobe (superior and inferior frontal gyrus, anterior cingulate gyrus). Thus, for instance, persistence shows a negative association with aromatase in the anterior cingulate gyrus, supramarginal gyrus, and lingual gyrus in men and a positive association with aromatase in the lingual gyrus in women; however, cooperativeness shows a positive association with aromatase in the anterior cingulate gyrus in men and a negative association with aromatase in the superior and inferior frontal gyrus in both sexes. These findings suggest that aromatase activity in some cortical structures may influence specific personality traits in humans, with sex-differentiated characteristics.

## Alterations of Brain Aromatase with Aging

There is limited information on the effects of aging on aromatase in the human brain. PET studies suggest a small age-associated decrease in aromatase expression in most brain regions.^29^ However, aromatase PET signal increases in the cortical white matter at older ages.^29^ Further, an immunohistochemical study detected a significant increase in aromatase immunoreactivity in all hippocampal regions of older (58–90 years old) compared with younger (34–43 years old) premenopausal women.^37^ The same study revealed an upregulation of ERα in the same hippocampal regions of older women, suggesting that the drop in ovarian estrogen production with menopause is compensated by an increase in estrogen production and signaling in the hippocampus.^37^ It is unknown whether a similar increase in aromatase and ERs occurs in other specific brain regions of postmenopausal women or in older men. However, in both men and women, brain levels of estradiol remain unaltered with aging, even if in men aging is associated with a decline in androgen levels in the brain.^76^ It is, therefore, plausible that brain aromatase activity, at least in some regions such as the hippocampus, could contribute toward maintaining local estrogen levels in older men and women. The preservation of the local estradiol levels in the aged brain may represent an endogenous protective response to maintain brain health, given that estradiol activates neuroprotective mechanisms in neurons and glial cells.^77^ However, further studies are still necessary to confirm this interpretation.

## Brain Aromatase Under Pathological Conditions

### Aromatase in ASDs

There is some speculation on the possible involvement of aromatase activity in ASDs. Single nucleotide polymorphisms within *CYP19A1* are associated with autistic traits and Asperger syndrome.^78^ In addition, a significant decrease in aromatase immunoreactivity was detected in a quantitative immunofluorescence analysis on postmortem samples from the frontal cortex (area 9 of Brodmann) of 12 autistic individuals, including males and females.^79^ Another study has reported a 38% reduction in *CYP19A1* mRNA levels in the middle frontal gyrus of 13 male ASD patients relative to controls, together with a 35% decrease in ERβ mRNA expression in the same region of the frontal cortex. Both changes in aromatase and ERβ expression were confirmed at protein level.^80^

*CYP19A1* mRNA expression in the frontal cortex is positively correlated with ERα and ERβ mRNA levels and with the mRNA levels of different ER cofactors,^80^ suggesting that ERs may be involved in the regulation of aromatase in this brain region. In this regard, it is important to consider that ERβ is not only a receptor for estradiol but also has affinity for the dihydrotestosterone (DHT) reduced metabolite 5α-androstane-3β,17β-diol.^81^ On the other hand, the expression of aromatase in the frontal cortex of ASD patients positively correlates with the expression of retinoic acid-related orphan receptor-α (RORA), which is also decreased in the brain of autistic patients.^79,82^ Interestingly, RORA is upregulated by estradiol through ERα and is downregulated by DHT through AR in human neuroblastoma cells. In turn, RORA transcriptionally regulates aromatase expression.^79^ Thus, it is tempting to speculate that the feedback mechanisms involved in hormonal regulation of RORA and aromatase are impaired in autistic patients. In addition, given the different effect of androgens and estradiol on RORA expression, it would be interesting to determine whether this molecule is involved in sex differences in ASDs, which affect predominantly males.

An implication of aromatase activity in autism would have important implications, considering that the enzyme is a target for endocrine disruptors and other environmental contaminants that may interact with ASD-associated genes during brain development.^83^ Indeed, as previously mentioned, aromatase is expressed in the developing human brain. Thus, by gestational week 17, aromatase immunoreactive cells are located at the proximity of the growing neuroepithelium of the ventricular and subventricular zones of the human cortex. Then, by gestational weeks 20–24, groups of immunoreactive aromatase cells are detected first in the cortical subplate and then in the parietal cortical plate. Later on, at perinatal and early postnatal periods, aromatase is expressed in glial fibrillary acidic protein immunoreactive glial cells in the cerebral cortex.^25^ Thus, aromatase activity could be potentially involved in neurodevelopmental disorders by the generation of estradiol, which may regulate specification, proliferation, or migration of neuronal and glial cells. Indeed, sex-specific deficits in cortical migration have been detected in aromatase knockout mice^84^ and estradiol reduces the impact of ASD-related mutations on cortical neurogenesis,^85^ suggesting that at least in animal models alterations in aromatase expression or activity during cortical development may cause permanent deficits in cortical function.

Another developmental factor associated with increased ASD risk that may alter aromatase expression is inflammation. Indeed, aromatase expression positively correlates with the expression of inflammatory markers in postmortem cerebellar samples of children who died between 1 and 9 years of age from pathologies associated with inflammatory conditions.^86^ This finding is relevant for the possible implication of local estradiol production in the brain in ASD, because the hormone is known to participate in the regulation of the development of cerebellar Purkinje neurons^87^ and because cerebellar alterations during infancy have been implicated in autism.^88,89^

### Aromatase and epilepsy

Changes in serum estradiol/progesterone ratio during the menstrual cycle or exogenous administration of estradiol are known to increase seizure susceptibility in women with epilepsy.^90,91^ In men, testosterone has mixed effects on epileptic seizures, because the reduced testosterone metabolite 3α-androstanediol decreases the frequency of seizures, whereas the aromatization of testosterone to estradiol increases seizures.^92^ This later effect may be mediated by the facilitating effect of estradiol on excitatory synaptic transmission and the suppression of inhibitory neurotransmission involved in seizures.^93,94^ Therefore, aromatase inhibitors, by decreasing estradiol levels, reduce seizures in men with epilepsy.^95^

Although estradiol is epileptogenic, it may also exert protective effects in epilepsy. Thus, the hormone decreases the latency to initiate seizures but reduces the duration of late-stage seizures in female rats treated with the epileptogenic drug kainic acid.^96^ In addition, estradiol protects gamma aminobutyric acid metabolism and prevents spine synaptic loss in hippocampal slices in which epileptogenic activity is induced with bicuculine.^97^ Further, aromatase inhibitors reduce seizures,^98^ but they increase hippocampal neuronal damage^15^ induced by kainic acid in rodents. These paradoxical effects of estradiol, which increases the frequency of epileptic seizures but at the same time decreases seizure severity and protects neurons from epileptic-induced excitotoxicity, could be explained by the rapid synaptic actions of the hormone, which potentiate excitatory transmission and suppress inhibition in neurons,^99,100^ in combination with the hormonal long-term transcriptional effects, which decrease excitotoxicity and oxidative stress, inhibit apoptosis, and promote synaptogenesis by acting on both glial and neuronal cells.^77^

Given these neuroprotective actions of estradiol, it would be important to assess the potential neurological long-term effects of aromatase inhibitors when considering their potential clinical use for the treatment of epilepsy. This information will be relevant, because histological studies have shown that most surviving parvalbumin inhibitory interneurons in the hippocampus of long-term epileptic patients express aromatase,^20^ suggesting that local estradiol formation in the epileptic hippocampus may still be able to regulate inhibition.

### Aromatase and stroke

Some *CYP19A1* polymorphisms are associated with an increased risk of stroke in both men and women, whereas other gene variants are associated with a decreased risk.^101^ The effects of these *CYP19A1* polymorphisms may be related with differences in systemic estradiol levels, because the hormone regulates numerous cardiovascular and metabolic parameters that affect the risk of stroke. However, central aromatase activity may also have some implications in the outcome of stroke.

Studies in rodents have shown an increased expression of the enzyme in reactive astrocytes in the regions of the neural tissue affected by secondary neurodegeneration after middle cerebral artery occlusion.^15,102^ This, together with the demonstration that estradiol generated by astrocytes after ischemic brain injury is neuroprotective,^103^ suggests a direct participation of aromatase activity in the outcome of stroke.

A similar increase in aromatase expression in reactive astrocytes has been detected after traumatic brain injury, where aromatase is induced by inflammatory signals and is neuroprotective by the local generation of estradiol.^15,104^ This suggests that central aromatase induction may be a general neuroprotective mechanism activated after an acute brain injury. Unfortunately, there is no information on whether aromatase expression is modified in the human brain after traumatic injury or stroke. Nevertheless, a recent study has reported an increase in serum aromatase levels 24 h after stroke in postmenopausal women.^105^ The functional significance of this finding is, however, unclear. Although aromatase concentration in serum has been shown to represent a good biomarker for some types of cancer,^106^ it is unknown whether it may be affected by modifications in aromatase expression in the brain.

### Aromatase and chronic neurodegenerative diseases

As observed for the risk of stroke, some aromatase gene variants also modify the risk of suffering chronic neurodegenerative diseases, such as Alzheimer's disease (AD). Thus, it has been reported that some *CYP19A1* polymorphisms, either *per se*^107–112^ or in combination with polymorphisms for other genes,^113,114^ are associated with increased AD susceptibility, whereas other aromatase gene polymorphisms may decrease AD risk.^115^

A direct evidence of changes in brain aromatase expression under neurodegenerative conditions in humans has been obtained in postmortem studies of multiple sclerosis lesions, where the enzyme is upregulated in astrocytes in male patients.^116^ Aromatase immunoreactivity is also increased in the hippocampus^21^ and the prefrontal cortex^38^ of male and female AD patients, compared with sex- and aged-matched controls. Further, aromatase immunoreactivity in the basal nucleus of Meynert, which sends cholinergic projections to the cerebral cortex and is important to maintain cortical function, is also increased in AD patients.^36^ In contrast, other brain regions of AD patients, including the hypothalamus, show decreased aromatase immunoreactivity compared with controls.^36^

The causes and significance of the changes in aromatase expression under chronic neurodegenerative conditions are not yet determined. As previously mentioned, animal studies suggest that increased aromatase expression after acute brain injury is neuroprotective by the local generation of estradiol. If these results are translatable to humans and to chronic neurodegenerative diseases, the enhanced expression of aromatase in multiple sclerosis lesions and in the hippocampus, the frontal cortex, and the basal nucleus of AD patients could represent an endogenous mechanism to protect the nervous system. On the other hand, aromatase upregulation in multiple sclerosis lesions and in the prefrontal cortex of AD patients is observed predominantly in astrocytes,^38,117^ which are known to actively participate in the neuroprotective actions of the enzyme by the generation of estradiol in animal experimental models.^16,103,117,118^

### Aromatase and sex differences in neurodegenerative diseases

The similarity in the expression of aromatase in the brain of men and women is intriguing, considering that robust sex differences are presented by the enzyme in the brain of other species, including laboratory rodents. This may be so because aromatase plays different functions in the sexual differentiation of the brain in rodents and primates. Indeed, although estradiol generated by brain aromatase is essential for rodent brain masculinization, it does not appear to play a major role in this process in humans and non-human primates.^119–121^ However, estradiol is important for male physiology^122,123^ and both testosterone and estradiol are involved in men libido.^124,125^ Further, the analysis of ERs and AR polymorphisms in transsexual populations suggests an interaction of AR and ERβ in the development of transsexuality in androphilic male-to-female transsexuals.^126^ However, in addition to estradiol, reduced metabolites of DHT are also ligands of ERβ^127^ and, therefore, the role of aromatase activity in the generation of sexual identity in men is still an unsolved question.

Aromatase activity could also be potentially involved in the generation of sex differences in neurodegenerative diseases. This is suggested by the studies showing that it is only in women that the plasmatic levels of the enzyme increase after stroke,^105^ whereas it is only in men that aromatase expression is increased in multiple sclerosis lesions.^116^ In addition, the association of *CYP19A1* gene polymorphisms with AD risk and mild cognitive impairment is more frequent in women than in men,^114,128^ further suggesting that aromatase activity may have a different effect on the neurodegenerative alterations of males and females.

Although not obvious sex differences in aromatase immunoreactivity have been detected in the brain of AD patients, studies in rodents suggest that aromatase activity may protect the female brain from AD pathology by increasing local estradiol levels but may have a negative impact on the male brain by decreasing local levels of testosterone.^129,130^ Whether this is the case in the human brain is unknown. Nevertheless, it is interesting to note that, compared with control individuals, estradiol levels decrease in the brain of women with AD, whereas testosterone but not estradiol levels decrease in the brain of men with AD.^76^

## Conclusions and Perspectives

The studies reviewed here indicate that aromatase is expressed in most, if not all, regions of the human brain, but in specific cellular populations within each region. Although some functional consequences of aromatase activity in the human brain have been explored, there are still numerous gaps in our knowledge of the role of the enzyme in the human CNS. For instance, although neurons are the main cell type expressing aromatase in the human brain, the enzyme is also expressed in astrocytes, oligodendrocytes, ependymal cells, and choroid plexus cells. These cells have numerous homeostatic, metabolic, and trophic functions in the brain; regulate cerebrospinal fluid formation and composition; generate myelin; control the blood–brain barrier; and participate in synaptic transmission and plasticity. However, we have very limited information on the role of local estradiol generated by aromatase in these cells, which are also involved in a variety of neurological alterations.

To our knowledge, nothing has been published about the local distribution and function of aromatase in the human spinal cord. In this regard, it would be important to confirm in humans the results of animal studies showing that aromatase is expressed by dorsal root ganglion neurons^41^ and that estradiol locally produced in the spinal cord and the thalamus by the enzyme is involved in pain transmission.^43–45^

Another CNS region where aromatase function needs to be better explored is the retina, where local estradiol synthesis may have a protective role.^49,131^ The role of aromatase activity also remains to be clarified in numerous brain regions; for instance, in the lateral habenula, where local estradiol synthesis by axonal afferent may participate in the modulation of neuronal circuits linking body homeostasis and motivation,^132^ or in the striatum/nucleus accumbens region,^29^ where testosterone and estradiol may influence motivation, anxiety-associated symptoms, reward cue processing, and drug abuse. It will be also important to clarify the role of aromatase activity in the vestibular nuclei, the cerebellum, and the olivo-cerebellar system,^22,29,133^ where estradiol locally produced by the enzyme may potentially be involved in the coordination or integration of sensory inputs with motor and cognitive processes.^134^ A better understanding of the role of aromatase activity in the different regions of the human brain will contribute toward clarifying the controversial cognitive effects of aromatase inhibitors used as breast cancer therapies^135,136^ and may also help to develop new treatments for specific brain pathological alterations based on drugs that modulate aromatase enzymatic activity or expression.

Another important question that remains to be elucidated is the role of aromatase activity during brain development. As discussed in previous sections, aromatase is expressed in the human fetal cerebral cortex^25^ and local production of estradiol by the enzyme is involved in the regulation of brain development in experimental animals. Further studies are necessary to determine its role in the developing human brain and whether its activity or expression during this sensitive period is altered by endocrine disruptor chemicals or by other pathological insults, such as maternal infections or by maternal chronic stress. Aromatase expression in the brain may be also altered by infections or inflammation during the early years of postnatal development, as it has been shown to occur in the cerebellum of male and female children between 1 and 9 years of age, where the enzyme expression is significantly increased in those children who experienced some form of inflammation proximate to death.^86^ If these changes in aromatase expression have an impact on brain development and early brain function, as animal experimentation studies suggest, they could play a role in the onset of developmental-associated brain disorders and in the generation of permanent functional deficits.

In addition to clarifying the role of aromatase activity in acute and chronic neurodegenerative diseases, affective disorders, and the brain aging process, further research is also needed to explore another very important clinical aspect: the role of the enzyme in brain tumors. Aromatase is expressed by human glioma cells,^32,137,138^ where it may play a role in tumor evolution by the generation of estradiol. Indeed, it has been found that in human glioblastoma multiforme (GBM) tissue the number of cells immunoreactive to aromatase and ERs decreased as the grade of tumor malignity increased.^139^ In addition, it has been recently reported that high expression of aromatase and ERα in human GBM is associated with significantly longer survival times of GBM patients, regardless of gender.^140^ These findings may pave the way for promising therapeutic perspectives for the control of glioma growth.

In conclusion, there are many unsolved questions and gaps in our knowledge on human brain aromatase that remain to be explored by future research. However, the limited available information that has been reviewed here suggests that the aromatization of androgens to estrogens by the human nervous tissue is involved in many more physiological and pathological processes than previously believed.

## Abbreviations Used

AD: Alzheimer's disease
AR: androgen receptor
ASD: autism spectrum disorder
CNS: central nervous system
DHT: dihydrotestosterone
ER: estrogen receptor
GBM: glioblastoma multiforme
GFAP: glial fibrillary acidic protein
Gr: granular layer
Hil: hilus
Mol: molecular layer
mRNA: messenger RNA
Or: Stratum oriens
PC: Purkinje cell layer
PET: positron emission tomography
Pyr: pyramidal layer
Rad: Stratum radiatum
RORA: retinoic acid-related orphan receptor-α
Wm: white matter
